# Single High Intensity Focused Ultrasound Session as a Whole Gland Primary Treatment for Clinically Localized Prostate Cancer: 10-Year Outcomes

**DOI:** 10.1155/2014/186782

**Published:** 2014-06-19

**Authors:** Ksenija Limani, Fouad Aoun, Serge Holz, Marianne Paesmans, Alexandre Peltier, Roland van Velthoven

**Affiliations:** ^1^Department of Urology, Jules Bordet Institute, 1 Rue Héger-Bordet, 1000 Brussels, Belgium; ^2^Université Libre de Bruxelles, 50 Franklin Roosevelt Avenue, 1050 Brussels, Belgium; ^3^Department of Data Management, Jules Bordet Institute, 1 Rue Héger-Bordet, 1000 Brussels, Belgium

## Abstract

*Objectives*. To assess the treatment outcomes of a single session of whole gland high intensity focused ultrasound (HIFU) for patients with localized prostate cancer (PCa). *Methods*. Response rates were defined using the Stuttgart and Phoenix criteria. Complications were graded according to the Clavien score. *Results*. At a median follow-up of 94months, 48 (44.4%) and 50 (46.3%) patients experienced biochemical recurrence for Phoenix and Stuttgart definition, respectively. The 5- and 10-year actuarial biochemical recurrence free survival rates were 57% and 40%, respectively. The 10-year overall survival rate, cancer specific survival rate, and metastasis free survival rate were 72%, 90%, and 70%, respectively. Preoperative high risk category, Gleason score, preoperative PSA, and postoperative nadir PSA were independent predictors of oncological failure. 24.5% of patients had self-resolving LUTS, 18.2% had urinary tract infection, and 18.2% had acute urinary retention. A grade 3b complication occurred in 27 patients. Pad-free continence rate was 87.9% and the erectile dysfunction rate was 30.8%. *Conclusion*. Single session HIFU can be alternative therapy for patients with low risk PCa. Patients with intermediate risk should be informed about the need of multiple sessions of HIFU and/or adjuvant treatments and HIFU performed very poorly in high risk patients.

## 1. Introduction

During the last decade, proactive screening for prostate cancer (PCa) led to a dramatic stage migration resulting in proportionally more men being diagnosed at early stages while the tumour is still organ confined [1]. Conventional established treatment options for organ confined PCa range from active surveillance to whole gland radical therapy [2]. Both radical prostatectomy (RP) and external-beam radiotherapy (EBRT) have undergone significant technical developments during the last decade and excellent long term cancer control data are available to support their clinical use [1]. However, these modalities are often associated with significant risk of treatment related complications that detrimentally affect quality of life [3, 4].

These facts have contributed to the development of “minimal invasive procedures” as an alternative option to standard therapies. Among these therapies, high intensity focused ultrasound (HIFU) emerged as a valid mini-invasive therapy for localised prostate cancer, using focused ultrasound to generate areas of intense heat to induce tissue necrosis. The ability of HIFU to achieve thermoablation of prostatic lesion was proven histologically on operative specimen [5], on MRI imaging [6], and on posttreatment biopsies [7, 8].

Oncological outcomes were first reported in 1995 [9] and 1996 [10] and subsequently the use of HIFU therapy has expanded. Nowadays, the available evidence on HIFU is comprised of case series with a significant overlap of patients among series. The median follow-up time is short with the longest series reporting data after a mean follow-up of 76.8 months [11, 12].

Another major problem is that most of the published case series included the results of retreatment in their efficacy rates. In the present single-centre study, we evaluated, at a median follow-up of 92 months, the oncological outcomes of single-session whole gland HIFU treatment of localised PCa in a unique case series of 110 patients treated between September 2001 and December 2012. The morbidity and urological after care were also analysed and discussed.

## 2. Patients and Methods

The study involved a cohort of 110 consecutive patients with clinically localized PCa primarily treated with whole gland HIFU at the Jules Bordet Institute between September 2001 and December 2012. Pooled prospectively collected data were retrospectively analyzed. Ethics approval in our institute covers the use of prospectively collected clinical information for clinical and prognostic studies.

Inclusion criteria were whole gland primary therapy with curative intent for localized PCa, prostate specific antigen (PSA) <20 ng/mL, clinical stage T1N0M0-T2N0M0, no previous radical therapy for PCa, and a follow-up >12 months. Baseline physical examination and PSA measurements were obtained for all patients. Extracapsular tumour extension and lymph node status were assessed using pelvic CT or MRI. Staging included a bone scan in patients with PSA ≥ 10 ng/mL, and laparoscopic lymphadenectomy was recommended in patients with PSA > 20 ng/mL. Exclusion criteria included clinically advanced PCa, evidence of metastatic or nodal disease on bone scan or cross-sectional imaging, prior significant rectal surgery, any contraindication for anaesthesia, and presence of prostatic calcification and cysts. All patients were unsuitable for surgery because of age, comorbidities, or patient refusal and were unwilling to undergo radiotherapy. All patients were treated by a single experienced surgeon, with Ablatherm HIFU devices (EDAP-TMS, Vaulx-en-Velin, France). From September 2001 to March 2006, patients were treated with the first commercially available device from Ablatherm (EDAP-Technomed, Lyon, France) and since April 2006 with Ablatherm Integrated Imaging (EDAP, Lyon, France). In our institution the upper volume limit for HIFU procedures is set to 40 cc and patients with prostates exceeding this threshold are offered androgen deprivation therapy (ADT) which was always discontinued at the time of therapy. All patients undergo a limited transurethral resection of prostate (TURP) at the end of the procedure, to prevent sloughing and acute urinary retention or prolonged need for indwelling catheter and to reduce the rate of urinary tract infection [5]. Complications were prospectively recorded and retrospectively graded according to the Clavien-Dindo score [13, 14]. Postoperatively, patients were followed with serial serum PSA determinations and DRE at 1, 3, 6, 12, 18, and 24 months and yearly thereafter. Oncological outcomes were evaluated using the D'Amico tumour recurrence risk group classification system [15]. Response rates were defined using the American Society for Therapeutic Radiology and Oncology (ASTRO)/Phoenix criteria (nadir + 2 ng/mL) (2005) [16]. Random systematic TRUS guided biopsies were offered only for a cause (phoenix criteria and/or suspicious DRE and/or PSA doubling time < 6 months). An individual PSA nadir was identified in each patient. PSA nadir was defined as the lowest PSA value reached during follow-up. Urinary functional outcomes were reported using physician reported rates. Stress incontinence was graduated according to Stamey into three grades [17]. Patients that were able to penetrate their partner without mechanical or pharmacological support were rated potent. Cause of death was identified from physician correspondence and all PCa specific deaths were verified. Overall QOL and costs were not reported in this study. The follow-up period was defined as the interval between HIFU treatment and last available monitoring data or the date of death. Only patients with complete data have been included in the final analysis (multivariate analysis, survival curves). A statistical analysis was performed with SPSS v.20 (IBM Corp., Armonk, NY, USA). Survival curves were based on the Kaplan-Meier method, and the log-rank test and a Cox regression model were used for univariate and multivariate analysis of the prognostic relevance of age, risk group, Gleason score, ADT, clinical stage, pretherapeutic PSA, and postoperative nadir PSA on biochemical recurrence, distant metastasis, and cancer specific survival. Actuarial survival rates were based on life table methods. A *P* value <0.05 was considered to indicate statistical significance.

## 3. Results

Baseline and tumour characteristics of the study population are summarized in Tables 1 and 2. Overall, a total of 110 patients (average age 76 years) were enrolled in this study with a median follow-up of 94 months. The mean (median) [range] PSA nadir was 0.55 ± 1.34 (0.71) [0–8.29] ng/mL and the mean (median) [range] time to achieve PSA nadir was 16.3 ± 8.2 (14.0) [4–46] weeks. A PSA nadir < 0.5 ng/mL was noted in 72/110 (65.5%) and 89/110 (80.9%) patients had a PSA nadir < 1 ng/mL. In spite of a good initial response to HIFU and a PSA nadir observation, two patients followed at distant centres were lost for evaluation and long term cancer control data were available for 108 patients (98.2%). During follow-up, 48/108 (44.4%) patients exhibited PSA elevation ≥2.00 ng/mL above nadir; they were offered a new set of bilateral biopsies, accordingly. Of the 35 (72.9%) patients who accepted control biopsy 22 were positive (62.9%). Of the 22 patients who experienced biochemical recurrence with a positive biopsy 12 patients were treated with a second HIFU session. Patients with negative biopsy or who refused biopsy were treated as follows: hormonal therapy (14 patients), second session HIFU (1 patient), and surveillance (11 patients). We evaluated retrospectively biochemical recurrence rate according to the definition of Stuttgart (nadir + 1.2 ng/mL): 50 (46.3%) patients experienced biochemical recurrence with a mean time to failure of 48 months.

**Figure 1:**
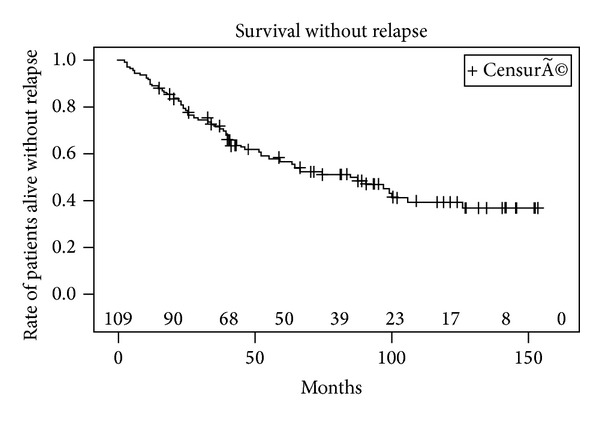
Kaplan-Meier curve of biochemical recurrence free survival using Phoenix definition (nadir + 2 ng/mL).

**Figure 2:**
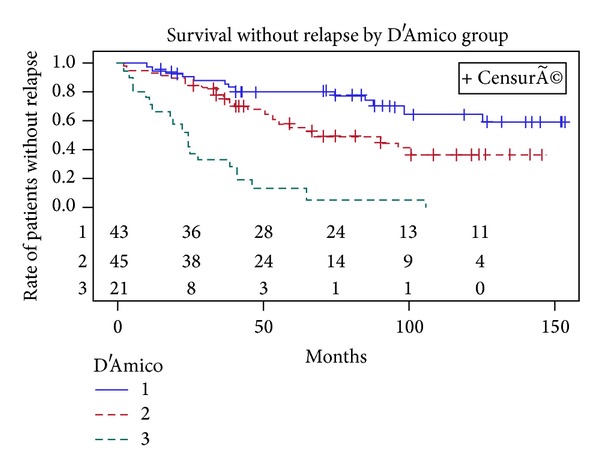
Kaplan-Meier curves of biochemical recurrence free survival using Phoenix definition (nadir + 2 ng/mL) according to D'Amico risk classification.

**Figure 3:**
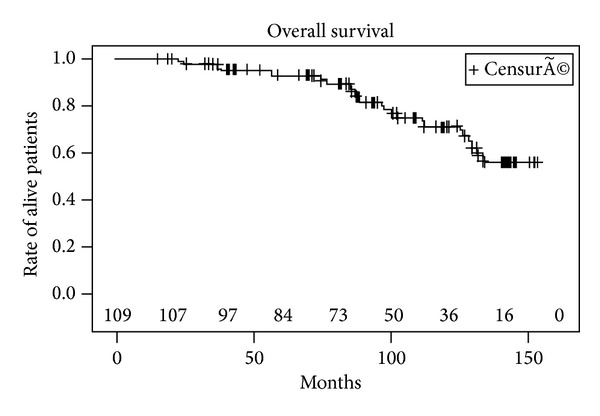
Overall survival.

**Figure 4:**
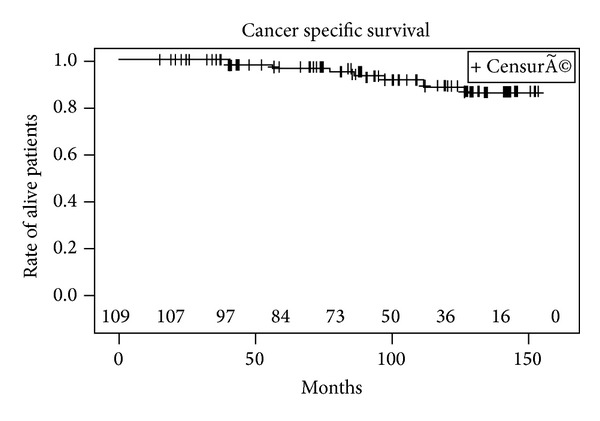
Cancer specific survival.

**Figure 5:**
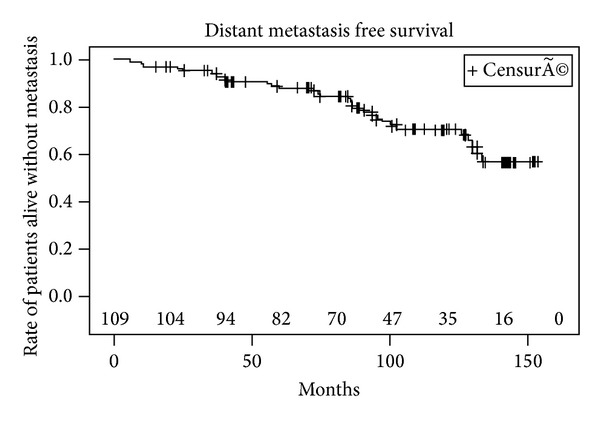
Distant metastasis free survival.

Metastases were detected in 10 patients (seven with bone metastases and 3 with lymph node involvement) after PSA relapse including 6 patients with a positive biopsy, 2 patients with a negative biopsy, and 2 patients who refused control biopsy. 20 patients died during follow-up, of which 8 patients died of cancer specific cause. The 5- and 10-year actuarial BRFS rates were 57% (CI 95%: 47–67%) and 40% (CI 95%: 29–51%), respectively (Figure 1). The median (range) time to oncological failure was 52 months (95% CI: 33–87 months). BRFS was significantly higher in patients in the low risk group compared to patients in the intermediate and high risk group (Figure 2). The 10-year estimated overall survival rate, cancer specific survival rate, and metastasis free survival rate were 72%, 90%, and 70%, respectively (Figures 3, 4, and 5). Preoperative high risk category, Gleason score, pPSA, and postoperative nadir PSA were independent predictors of oncological failure in univariate and multivariate analysis (Table 3).

The incidences of the most frequent complications were reported in Table 4. Regarding grade 1 and grade 2 complications, 27 patients (24.5%) had self-resolving hematuria and LUTS, 7 patients had storing LUTS treated by anticholinergics (6.4%), 4 patients had chronic pelvic pain, and 20 patients (18.2%) had urinary tract infection. A grade 3a complication occurred in 20 patients (18.2%) who had acute urinary retention. A grade 3b complication occurred in 26 patients who had bladder outlet obstruction or urethral stricture managed by optical urethrotomy and in one man (0.9%) who had developed a rectourethral fistula managed surgically. No patient presented any grade 4 or died from the procedure. Urinary functional outcomes were reported using physicians reported rates. Grade 1 stress urinary incontinence was reported in 8 patients (7.4%) and 5 patients (4.6%) had grade 2 stress urinary incontinence. The long term pad free continence rate was 87.9%. In preoperatively potent patients (*n* = 68), 7 men (10.3%) had documented post whole gland ablation erectile dysfunction (ED), 47 men (69.1%) had erections satisfactory for sexual intercourse with (*n* = 16) or without pharmacotherapy (*n* = 31), and data were lacking in 14 patients. The mean age of patients with postoperative ED was 77.3 years. If we consider the 14 patients with lacking data to have ED after treatment, the ED rate for this cohort post treatment would be 30.8% (21/68) and 45.6% (31/68) had erectile function sufficient for penetration without pharmacotherapy.

## 4. Discussion

Despite the fact that HIFU has been used in PCa for over 15 years [10], the European Association of Urology, the American Urological Association, and the National Comprehensive Cancer Network do not recommend the routine use of HIFU in the primary treatment of PCa. Disapproval is mainly due to the absence of prospective randomised controlled trials comparing HIFU with conventional treatment options and to the paucity of long term oncological follow-up data. Long term results on the efficacy and safety of HIFU have rarely been reported [12–20]. In the present study, only patients with a minimal follow-up of 1 year were included. With a median follow-up of 92 months, a well-founded oncologic, safety, and functional evaluation of whole gland ablation HIFU as a therapy for PCa was possible.

Assessment of oncologic efficacy was performed by serial PSA testing and random systematic TRUS guided biopsies were offered only for a cause in order to minimise burden on the patient. Furthermore, performance of systematic biopsies in all patients may increase the cancer detection rates during follow-up but the clinical implication of such a protocol is unknown because it may simply reveal small foci of low grade low volume PCa. In our opinion, the only valid endpoint with a follow-up >1 year is the PSA nadir and the biopsy should be offered routinely in case of a PSA elevation, a suspicious lesion on DRE and/or multiparametric MRI and/or contrast ultrasound. As a surrogate, although PSA testing is accepted as a valid outcome in standard therapies, the clinical utility of PSA kinetics in HIFU is yet to be determined. We acknowledge that the use of Phoenix criteria is a shortcoming of the present analysis and the need for a specific definition of treatment failure to evaluate the clinical outcome after HIFU.

In our series, there was a 95.4% decrease in PSA levels from baseline and a PSA nadir <0.5 ng/mL was noted in 65.5% of patients which indicate successful ablation of the prostate. The early achievement (between 3 and 6 months) of a PSA nadir not only provides immediate feedback on treatment efficacy but also identifies quickly patients with residual cancer. This rapid proof of a response to the treatment provides also stringent information about potential cure. The maintenance of a PSA < 0.5 ng/mL was noted in 50% (36/72) of these patients at 10 years of follow-up; only 12.5% (9/72) of these patients experienced biochemical failure according to the Phoenix criteria during the follow-up. The PSA nadir was, in our study, an independent predictor factor of oncologic failure (cancer specific survival, BRFS) in multivariable analysis. The PSA nadir, in most contemporary series, was found to be a surrogate for predicting treatment failure [21, 22]; however, a cut-off has not been yet standardized [23]. Furthermore, PSA nadir correlated strongly, in these studies, with preoperative baseline PSA and prostate volume. In our study, preoperative PSA was also an independent predictor factor to estimate the risk of treatment failure. This could be explained by the high likelihood of extraprostatic disease with increasing PSA level. Larger prostate volume remnants will produce a greater amount of PSA. In our study, cytoreductive ADT and the high percentage of TURP had led to small prostate remnants (mean 14 cc) and the prostate volume was not found to be an independent predictor of treatment failure.

A durable response was also seen in 68% of patients with low risk disease at 10 years of follow-up. All patients classified as D'Amico high risk experienced biochemical recurrence and were treated by second HIFU, salvage treatment, or palliative ADT. Intermediate risk disease presented an unacceptable risk of biochemical relapse when treated with a single session of HIFU (BCR free survival of 40% at 10 years) but with HIFU retreatment a biochemical recurrence free survival of 53% was achieved at 10 years of follow-up. HIFU delivered with intention to treat performs very poorly in high risk groups and should be indicated only for patients with localised PCa at low and intermediate risk of progression. Patients with intermediate risk of progression should be informed about the further need of multiple sessions of HIFU and/or adjuvant treatments. Some authors advocated HIFU plus ADT as an alternative to ADT plus EBRT in high risk PCa [24]. ADT, in the presented series, was not shown to be an independent predictor of oncologic outcomes but it was given for a short period of time, in a small percentage of patients, and according to prostate volume and not to the risk of progression. In routine practice, these prognostic factors should serve to the establishment of nomograms and would be useful for the clinician in informing patient regarding the likelihood of requiring salvage treatment. HIFU as a minimal invasive procedure appears to be one of the most attractive options for the treatment of localized PCa because of its low morbidity rates [21]. In our study, early self-resolving LUTS due to sloughing of necrotic tissue from the coagulated gland were the most common complications. Elimination of debris through micturition and swelling of the prostate due to thermal injury were the principle cause of acute urinary retention. Another important complication linked also to necrotic tissue is the high risk of urinary tract infection. Bladder outlet obstruction and urethral stricture are two long term complications occurring in 3.6% to 24.5% of patients [12–22] (24% in our study). The introduction of real time monitoring had dramatically decreased the incidence rate and the severity of these complications. Our study shows a good gastrointestinal tolerance with no late onset toxicity and no rectal toxicities were reported when real time monitoring was introduced. An urethrorectal fistula occurred in only one patient in a retreated gland with no monitoring of the rectal wall. Experience with the procedure, the addition of a cooling system, and safety monitoring with good margins has dramatically decreased the incidence of fistula which now ranges between 0.5 and 1.2% [21]. The procedure could possibly be delivered in an ambulatory care setting; the long stay of 4 days in our series is related to local reimbursement practice, preoperative anaesthetic evaluation, and transurethral partial resection of the prostate. Stress urinary incontinence occurred in 12% of patients, which is comparable to the rates reported in the literature [22–25].

In the current study, 30.8% of initially potent patients exhibited erectile dysfunction after HIFU therapy witch is in the range of 20–49.8% of the rates reported in the literature [26]. Generally the preservation of the lateral edges of the prostate permits to spare erectile function but should always be balanced with the risk of oncologic failure. Recently, we have reported better stress urinary incontinence and erectile function rates with hemiablation HIFU but validated questionnaire and further experience is therefore needed to confirm this important conclusion [27].

To our knowledge, our study is the first to report on a unique cohort of patients primarily treated by a single session of whole gland ablation HIFU for a clinically localised PCa with a median follow-up of 92 months. Moreover, a very limited number of patients were lost to follow up (1.8%) and all patients were treated by the same experienced surgeon.

Meanwhile we acknowledge several limitations to our study. First, the safety, functional, and oncologic outcomes are the results of a single centre with a long experience with whole gland HIFU and cannot be generalized. The outcomes could be variable in less experienced hands because HIFU is a dynamic therapy with real time feedback which is difficult to master while assessing quality control [28]. Second, the study reported retrospectively on a small cohort of patients. Well-designed, multicenter, prospective, and randomized controlled studies are required to assess collateral damage, functional and oncologic outcomes. Third, technological improvements (real time monitoring) and changes in surgical protocol (TURP, prophylactic antibiotics) may have confounded some of the outcome analyses. Fourth, a direct comparison of outcomes with other conventional PCa therapies is not possible due to differences in selection criteria, study design, and adjuvant/salvage therapies. Fifth, bias could have been introduced by including heterogeneous patient population with variations in prognostic factors and neoadjuvant ADT but the latter is unlikely to confound PSA outcomes especially when it is given for a short duration and the follow-up is long. In addition, neoadjuvant ADT was not a significant predictor of survival in the univariate analysis.

## 5. Conclusions

Our study, with a median follow-up of 92 months, has provided evidence that HIFU can be an alternative treatment for patients with low risk localised PCa who are not eligible or refuse conventional therapy. Patients with intermediate risk should be informed about the further need of multiple sessions of HIFU and/or adjuvant treatments. HIFU delivered with intention to treat performed very poorly in high risk patients, and those patients should probably be offered a more conventional treatment, such as radical surgery or radiation therapy. Patients should also be informed that whole gland HIFU ablation, though being a mini-invasive energy based modality, has a side effect profile that cannot be negligible and is offered in a study setting. Preoperative high risk category, Gleason score, pretherapeutic PSA, and postoperative nadir PSA were independent predictors of oncological failure and should serve the establishment of nomograms and could be useful for the clinician in informing patient regarding the likelihood of requiring salvage treatment.

## Figures and Tables

**Table 1:** Baseline and tumour characteristics of 110 patients with localized prostate cancer who were treated by a single session of high intensity focused ultrasound.

Mean age, years [range]	76.1 ± 6.2 [61–86]
Mean preoperative PSA, ng/mL [range]	12.1 ± 4.1 [0.55–49.0]
Mean prostate volume, mL [range]	29.3 ± 6.0 [18–39]
Hormone, *n* (%)	
Yes	37 (33.6)
No	73 (66.4)
Gleason score, *n* (%)	
≤6	69 (62.7)
=7	24 (21.8)
≥8	17 (15.5)
Stage, *n* (%)	
T1	51 (46.4)
T2	59 (53.6)
D'Amico risk group∗, *N* (%)	
Low	40 (36.4)
Intermediate	49 (44.5)
High	21 (19.1)

*Risk group based on D'Amico definition (according to Stage, Gleason, and PSA).

**Table 2:** Intraoperative and postoperative results with high intensity focused ultrasound.

Anesthesia used	
Spinal anesthesia	109
General anesthesia	1
Ablatherm device	
EDAP-Technomed Ablatherm	59
Ablatherm Integrated Imaging	51
Preoperative complications	0
Concomitant TURP, *n* (%)	
Yes	79 (71.8)
No	31 (28.2)
Hospital stays in days, median (range)	4 (2–7)
Catheterization time in days, median (range)	6 (2–30)
Postoperative prostatic volume in mL, median (range)	14 (6–22)
Time to PSA nadir in weeks, mean (range)	16.3 ± 8.2 (4–46)
PSA nadir in ng/mL, mean (range)	0.55 ± 1.34 (0–8.29)
PSA < 0.5 ng/mL, *n* (%)	72 (65.5)
PSA 0.5–1 ng/mL, *n* (%)	17 (15.5)
PSA 1–4 ng/mL, *n* (%)	19 (17.2)
PSA > 4 ng/mL, *n* (%)	2 (1.8)
Follow-up in months, median (range)	94 (13–139)
Lost to follow-up, *n* (%)	2 (1.8)

**Table 3:** Univariate and multivariable analysis of factors affecting biochemical recurrence free survival in 108 patients.

	Univariate			Multivariate		
	OR	95% CI	*P* value	OR	95% CI	*P* value
Age (per 10 years)	0.95	0.51–1.76	0.87	—	—	—
Gleason score (<7; ≥7)	2.01	1.15–3.51	0.01	1.79	1.04–3.08	0.01
Prostate volume (per 10 mL)	1.29	0.74–2.22	0.37	—	—	—
Pretherapeutic PSA (per ng/mL)	1.06	1.04–1.09	<0.0001	1.08	1.05–1.11	<0.0001
PSA nadir (per ng/mL)	1.92	1.58–2.33	<0.0001	1.87	1.54–2.27	<0.0001
ADT before HIFU (yes v/s no)	1.56	0.92–2.62	0.1	—	—	—
D'Amico risk group (low v/s intermediate)	1.64	0.83–3.24	0.15	—	—	—
D'Amico risk group (low v/s high)	3.36	1.61–7.03	0.001	2.9	1.43–5.89	0.003

OR: odds ratio; CI: confidence interval; PSA: prostate-specific antigen; ADT: androgen deprivation therapy; HIFU: high intensity focused ultrasound.

**Table 4:** Adverse events.

	EDAP-Technomed Ablatherm (*N* = 59)	Ablatherm integrated imaging (*N* = 50)
Acute urinary retention (%)	15 (25.4%)	5 (10.0%)
Urinary tract infection (%)	16 (27.1%)	4 (8.0%)
Lower urinary tract Symptom (%)	16 (27.1%)	11 (22.0%)
Chronic pelvic pain (%)	4 (6.8%)	0 (0.0%)
Bladder outlet Obstruction (%)	18 (30.5%)	8 (16.0%)
Recto-Urethral Fistula (%)	1 (1.7%)	0 (0.0%)
